# The Necessity of Complete Extirpation of Tumors and the Importance of Rapid Cicatrization of the Wound

**Published:** 1895-11

**Authors:** Frederick Holme Wiggin

**Affiliations:** New York; Visiting Surgeon to the New York City Hospital (B. I.), Gynæcological Division; 55 West 36th Street


### Southern Medical Record.

A MONTHLY JOURNAL OF MEDICINE AND SURGERY.

VOL. XXV. ATLANTA, GA., NOVEMBER, 1895. No. 11.

### Original Articles.

### THE NECESSITY OF COMPLETE EXTIRPATION OF TUMORS AND THE IMPORTANCE OF RAPID CICATRIZATION OF THE WOUND.

BY FREDERICK HOLME WIGGIN, M. D.,

Visiting Surgeon to the New York City Hospital (B. I.), Gynæcological Division.

Neoplasms occur with greater frequency in the female than in the male subject. Statistics show that the breast, next to the uterus, is the most usual site of these morbid changes—seventeen per cent. of all cases occurring in the former, and nineteen per cent. in the latter. Williams found in a collection of 13,824 primary neoplasms, 2,397 cases in which the female breast was affected, while only 25 similar cases were found to exist in males. We may, therefore, with propriety limit ourselves in considering and answering the questions of the necessity of complete extirpation of tumors and the importance of the rapid cicatrization of the wound to the neoplasms of this region in the female. It may be well once more to call attention to the fact that malignant growths occur in all parts of the body more frequently than do those which are more benign. According to Williams, 95 per cent. of all breast neoplasms are malignant. This preponderance of malignant tumors, coupled with the fact that at times benign neoplasms take on malignant characteristics, proves at once the fallacy of the widespread

*Read at the Twelfth Annual Meeting of the New York State Medical Association, October 16th, 1895, as a part of the discussion on Surgery.

belief which, contrary to the teaching of Gouley and others, still continues to exist in the minds of many general practitioners, that as long as a tumor remains quiescent it is unwise to remove it. This idea undoubtedly originated in the dread which surgical procedures, undertaken for the relief of these morbid conditions, inspired in the minds of both patient and physician, partly on account of the high rate of mortality which formerly followed them, and partly because they seldom afforded even temporary relief to the sufferer. We can hardly wonder that these patients, failing to receive encouragement that their condition could be materially benefited by drugs or operative measures, should either do nothing or should, in their despair, turn towards the charlatan in the vain hope that possibly he could in some degree make good his promises of cure.

While, undoubtedly, this was a true statement of the results of the treatment employed by physicians a few years since, it is by no means a fair representation of the case to-day, and it is the purpose of this paper to show why the older surgeons so often failed in their treatment of this class of cases, and the methods by means of which so much better results are obtained with certainty to-day and the surgeons enabled to hold out hope, if not of cure, of long periods of freedom from the disease. The most frequent cause of death following these operations in the past was septic infection; but thanks to the discoveries of Pasteur, and their adaptation to surgical practice by Lister, and the changes which have finally ended in the aseptic technic of the present day, the mortality following these operations has been reduced from 25 per cent. to practically none.

Said Dr. J. W. S. Gouley in the course of a discussion on tumors before this Association in 1888, "From a scientific standpoint, it cannot be said that malignant neoplasms are ever cured, since it is known that their tendency to recur is strong and that the period of their recurrence is indefinite." But it is a well-established fact that after three years have elapsed, the tendency to recurrence is slight, and for the purposes of this discussion this period of immunity will be considered as the test of success of the methods employed by the surgeon. Formerly, when it was customary to remove only the tumor, the results were unsatisfactory, and few surgeons succeeded in giving their patients this period of immunity. If we accept the cellular theory of the genesis of neoplasms, it can be readily understood, as has been pointed out by Williams, that these lesions are seldom limited to their starting point. Sir Astley Cooper, in the course of his lectures on surgery, published in 1839, page 386, said, "I would observe that the scirrhous tumor is not all the disease; there are roots which extend to a considerable distance, and if you would remove the tumor only and not the roots, there will be little advantage from the operation." Again the same author in his lectures on surgery published in 1821, Lecture XXI., page 251, in describing the technic of the operation of excision of a mamma containing a malignant tumor, said, "Let both the incisions be carried down to the pectoral muscles and dissect out the tumor close to the latter, so as to lay it completely bare, removing even the fascial covering, for if this be not minutely attended to, there will be a very great probability of the disease returning, or I may say with propriety, remaining." Again, "The glands in the axilla, if enlarged, are now to be cautiously removed, together with the intervening substance, as leaving the latter would be the future cause of a similar disease being produced." In 1866, Charles H. Moore, F. R. C. S., in his paper entitled, "On the Influence of Inadequate Operations on the Theory of Cancer," *Medico-Chirurgic Transactions*, vol. L, page 245, said, "When any texture adjoining the breast is involved in, or even approached by the disease, that texture should be removed with the breast. This observation relates especially to skin, to lymphatics, to much fat and to pectoral muscles. The attempt to save the skin which is in any degree unsound is, of all errors, the most pernicious, and whenever its condition is doubtful, that texture should be freely removed. In the performance of the operation, it is desirable to avoid not only cutting into the tumor, but also seeing it; no actually morbid texture should be exposed, lest the active microscopic element in it be set free and lodge in the wound. Diseased axillary glands should be taken away by the same dissection as the breast itself, without dividing the intervening lymphatics; and the practice of first roughly excising the central mass of the breast and afterwards removing successive portions which may be of doubtful soundness, should be abandoned. Only by deliberately reflecting the flaps from the whole mamma and detaching it first at its edge, can the various undetected prolongations of the tumor and outlying nodules be included in the operation. To parts not capable of removal, it is desirable to apply chloride of zinc."

It would appear that Sir Astley Cooper was the first to recognize the fact that the disease was not confined wholly to the mamma where it originated, that in cases of scirrhous tumors of this region, the axillary, infra and supra, clavicular gland early become infectedand enlarged and should be removed, that the incision should be made wide of the disease and down to the pectoral muscle; and he advocated the removal in all cases of the pectoral fascia. He called attention to the fact that the reappearance of the disease is often not a true recurrence but a "remaining" or continuance of the disease. In other words, the operation has been an incomplete and, therefore, unsuccessful one when, after a short interval, the disease reappears locally and cannot be considered a reinfection. Had he left out the words "if enlarged" in his advice to clear out the axilla, little would have been left for the so-called originators of the modern, complete operation to discover. In these views, Moore coincided, reiterating the importance (1) of the complete removal of the diseased organ, (2) of the necessity of cutting so wide of the disease that none of it should appear in the course of operation and (3) the removal in one mass of all the tissues (including a liberal margin of apparently healthy skin).

Notwithstanding this sound and brilliant teaching, surgeons continued to perform partial operations only. Dr. Curtiss in the course of his article entitled "The Cure of Cancer by Operation," *Medical Record*, February 24th, 1894, said, "Gross found in those cases subjected to operation in which the site of recurrence is noted that in 96 cases operated upon without touching the glands, the disease reappeared in the cicatrice or vicinity alone in 48 per cent., in the axillary glands alone in 20 per cent., and in both in 32 per cent., returning in the glands in 52 per cent. of the cases. On the other hand, in 313 cases in which the axilla was cleared, the percentage of recurrences was 75 locally, twelve in the glands, and thirteen in both; a reduction of the glandular recurrences from 52 per cent. to 25

per cent." These statistics showed the importance of including the axillary glands in the tissues to be removed. But Küster was probably the first to prove that the glands may be infected and, therefore, a source of continuation of the disease before they begin to enlarge. Volkman called attention to the fact that the loose areolar tissue between the glands and the pectoralis major muscle contains glandular offshoots and lymphatics which, in malignant cases, are diseased. Heidenhain proved that these lymphatics may adhere to the fascia without penetrating it, and that there is not free communication between them and the lymphatics of the muscle. With the recognition by Volkman, Banks, Gross, Bull, Dennis and others of the importance of these views and their practical adoption, came a marked diminution in the percentage of recurrences or, more properly speaking, continuance of the disease, the cures amounting to about 20 per cent. Bull has lately, in the course of an article entitled, "The Cure of Carcinoma of the Breast by Radical Operation, *Medical Record*, volume 46, page 225, stated his individual results to be 26.6 per cent.

Dennis, in his article entitled, "Recurrence of Carcinoma of the Breast," read before the American Surgical Association in 1892, stated that 25 per cent. of the cases of carcinoma of the breast operated upon by him had passed the three-year limit without recurrences, and subsequently, at a recent meeting of the Litchfield County (Conn.) Medical Association, "that in a series of fifteen of his last cases, the results show 83 per cent. of recoveries," and in those cases which were operated on by him within six months of the appearance of the disease, "cures had been secured in all cases."

Volkman, in a few of his worst cases, excised the pectoral muscles, as well as the other tissues ordinarily removed by him. This addition to his technic was followed by results more satisfactory than his previous ones, the disease reappearing in only 35 per cent. of these cases against 60 per cent. in those cases in which the muscles were left intact. Halsted, acting on this suggestion, has for some time included this procedure in his operations for the removal of carcinomatous mammæ with apparently wonderful results, he stating the so-called recurrences to be only 6 per cent. in the cases operated on by him from June, 1889, to January, 1894, but in many of these cases sufficient time had not elapsed when his paper was written, to make the test either a fair or satisfactory one.

Professor W. H. Welch, in the course of the discussion previously alluded to, held before the Litchfield Medical Association, confirmed the necessity of this addition to the technic, for he said, "that frequently microscopical examinations of the pectoral muscles in cases in which there was no appearance of cancerous deposit, showed a plugging up of a lymphatic by a group of several cancer cells; therefore," he said, "the rule for cutting wide of the disease has the very best foundation in microscopical examination." He also added that "a carcinoma was always unquestionably a malignant tumor, but microscopical examinations of sarcomata did not allow one to speak with the same assurance as to the malignancy of these tumors. Thus, sarcomata which were made up of small, round cells with very little basement substance, were most malignant tumors; on the other hand, the spindle-cell sarcomata might be localized and never give rise to metastasis.

By a complete operation, then, is meant one that not only removes the entire mamma and all the skin that surrounds it, but the axillary glands and those contained in the infra and supra clavicular space as well as those that lie between the edges of the pectoralis major and deltoid muscles, the loose areolar tissue underlying the gland, and the fascia covering the great pectoral muscle; and if more than six months has elapsed since the detection of the primary neoplasm, the pectoral muscle as well, the incisions being carried wide of the diseased tissues, which are removed in one mass, thus avoiding the danger of dissemination of cancerous fragments in the wound, the smallest particle of which is sufficient to form a nucleus for recurrence or continuation of the disease.

Halsted, Mayer, and Curtiss report that but little of deformity and functional disturbance follows the extirpation of the pectoral muscles, major and minor.

There can be at this time little doubt that the reason of the failure of the older sugeons to obtain satisfactory results was due, in the first place, to septic infection, and in the second place, to late and incomplete operations. The remedy seems at present to be largely in the hands of the general practitioner, as well as in those of the surgeon, for, as we have seen, much depends on the promptness with which the operation is advised and performed. Too much stress canot be laid on the importance of the complete extirpation of neoplasms, for upon the thoroughness with which this is accomplished, depends the cure or interval of immunity from the disease. To the questiou of what importance is the rapid cicatrization of the wound, it may be answered that while it is of consequence that every wound should heal as rapidly as possible, in the cases we have been considering, it should be deemed a matter of secondary importance to the free removal of the tissues adjacent to the diseased structures. The rapid healing of the wound may be promoted by skin grafting according to the method of Thiersch, or by Schede's method of the organization of the blood clot.

With a better understanding on the part of the general practitioner of the necessity for the early extirpation of all neoplasms, especially of those of the mammary region, and on the part of the surgeon of the vital importance of the complete operation, it seems reasonable to expect that in the near future the surgeon's art will triumph over this mortal foe of womankind, and that a reasonable hope of cure can be confidently offered those afflicted with this most malignant of diseases.

*55 West 36th Street.*

---

CHLOROFORM IN PARTURITION.—Dr. Bedford Brown, of Alexandria, Va., says it is the extraordinary development of the vasomotor and circulatory system that gives immunity to pregnant women, at parturition, from the evil effects of chloroform. That this immunity does exist, less than forty deaths in the world, to date, from this cause, is proof enough. The doctor uses chloroform to relieve pain at any stage; to shorten labor by relaxing the cervix and perineum. He does this without disturbing the uterine contractions. He also thinks that chloroform tends to prevent post-partum hemorrhage and lacerations. He gets great satisfacticn from the use of chloroform in retained placenta, with contracted and rigid os and perineum. All parts are relaxed so that extraction is a matter of routine only.—*Med. and Surg. Reporter.*

